# Corrigendum to “The Studies of Chlorogenic Acid Antitumor Mechanism by Gene Chip Detection: The Immune Pathway Gene Expression”

**DOI:** 10.1155/2015/538539

**Published:** 2015-03-16

**Authors:** Tian Yi Kang, Hua Rong Yang, Jie Zhang, Dan Li, Jie Lin, Li Wang, XiaoPing Xu

**Affiliations:** ^1^West China School of Pharmacy, Sichuan University, Chengdu, Sichuan 610041, China; ^2^Jiuzhang Biochemical Engineering Science and Technology Development Co., Ltd., Chengdu, Sichuan 610041, China

In the paper titled “The Studies of Chlorogenic Acid Antitumor Mechanism by Gene Chip Detection: The Immune Pathway Gene Expression,” Journal of Analytical Methods in Chemistry Volume 2013 (2013), Article ID 617243, 7 pages, doi: 10.1155/2013/617243, some errors occurred in the “Disclosure” and “Acknowledgments” sections and they should be corrected as follows.

